# Correlation of ethnicity with breast density as assessed by Quantra™

**DOI:** 10.1186/bcr2951

**Published:** 2011-11-04

**Authors:** D Tzias, S George, L Wilkinson, R Mehta, C Lobo, A Hainsworth, A Sharma

**Affiliations:** 1 St George's Hospital, London, UK

## Introduction

It is widely accepted that there is an association between mammographic density and breast cancer risk [1]. Various studies have examined relationships between ethnicity and breast density patterns using the Wolfe classification system [2], with a view to investigating potential breast cancer risk. Quantra™ is a volumetric assessment tool, which allows reproducible objective measurement of mammographic breast density, eliminating inter-observer variability. Our study sought to investigate the correlation between ethnicity and breast density using Quantra™ measurements.

## Methods

The Quantra™ value was recorded from the mammograms of symptomatic breast patients at St George's Hospital, London over a 6-month period. We compared three distinct ethnic groups; Black (African and Afro-Caribbean), Asian (Indian subcontinent) and White (Caucasian). Mean Quantra™ values were calculated for each group and the Kruskal-Wallis test was applied.

## Results

A total of 428 patients were included in the study. Mean breast density values for the three ethnic groups were as follows: Black, 24.31%; Asian, 21.94%; White, 24.74%. *P *= 0.0046 (Kruskal-Wallis).

## Conclusion

There is a statistically significant difference between the objectively measured breast densities of these three ethnic groups. This is of relevance to the assessment of breast cancer risk.
